# Chloroquine Suppresses Colorectal Cancer Progression via Targeting CHKA and PFKM to inhibit the PI3K/AKT Pathway and the Warburg Effect

**DOI:** 10.7150/ijbs.101921

**Published:** 2025-01-27

**Authors:** Yanqing Liu, Yongping Zhu, Liwei Gu, Kexin Li, Ang Ma, Li Liu, Yuqing Meng, Junzhe Zhang, Shengnan Shen, Qiaoli Shi, Dandan Liu, Xinwei Zhang, Shujie Zhang, Xin Chai, Peng Gao, Jiale Xing, Yaxu Wang, Honglin Chen, Rui Liu, Qingfeng Du, Haitao Liu, Lingyun Dai, Jigang Wang

**Affiliations:** 1 State Key Laboratory for Quality Ensurance and Sustainable Use of Dao-di Herbs, Artemisinin Research Center, and Institute of Chinese Materia Medica, China Academy of Chinese Medical Sciences, Beijing 100700, China.; 2 Department of Traditional Chinese Medicine, Peking Union Medical College Hospital, Peking Union Medical College, Chinese Academy of Medical Sciences, Beijing 100730, China.; 3 Key Laboratory of Computational Chemistry-Based Natural Antitumor Drug Research & Development, Liaoning, Shenyang Pharmaceutical University, Shenyang 110016, China.; 4 State Key Laboratory of Antiviral Drugs, School of Pharmacy, Henan University, Kaifeng 475000, China.; 5 College of Animal Science and Technology, Henan Agricultural University, Zhengzhou 450002, China.; 6 School of Traditional Chinese Medicine and School of Pharmaceutical Sciences, Southern Medical University, Guangzhou 510515, Guangdong, China.; 7 State Key Laboratory of Bioactive Substance and Function of Natural Medicines, Institute of Medicinal Plant Development, Chinese Academy of Medical Sciences, Peking Union Medical College, Beijing 100193, China.; 8 Department of Critical Care Medicine, Guangdong Provincial Clinical Research Center for Geriatrics, Shenzhen Clinical Research Centre for Geriatrics, Department of Nuclear Medicine, Shenzhen People's Hospital (The First Affiliated Hospital, Southern University of Science and Technology; The Second Clinical Medical College, Jinan University), Shenzhen, Guangdong 518020, China.

**Keywords:** Colorectal cancer, Chloroquine, PI3K/AKT pathway, Warburg effect

## Abstract

Colorectal cancer (CRC) is the second leading cause of cancer-related death worldwide and has become a recognized global health problem. Therefore, the search for new anti-CRC agents or the exploration of new effective drug targets for CRC therapy is urgent. Chloroquine (CQ) is a widely-used antimalarial drug and has shown anti-proliferative effects in CRC. However, the underlying mechanisms are not well understood, particularly as the direct targets of CQ have not been identified. In this study, choline kinase alpha (CHKA) and ATP-dependent 6-phosphofructokinase, muscle type (PFKM) were identified and verified as the binding targets of CQ. CQ specifically binds to CHKA, inhibits its expression and enzymatic activity, and downregulates the downstream phosphorylation of PI3K and AKT, thereby suppressing tumor cell proliferation and inducing apoptosis. CQ also binds to PFKM and inhibits its expression and activity, thereby blocking the Warburg effect. In addition, the downregulation of CHKA can decrease the expression of PFKM and inhibit its activity, thereby blocking the Warburg effect. These observations shed new light on the antitumor mechanisms of CQ and provide new evidence for the close relationship between the PI3K/AKT signaling pathway and the Warburg effect, providing new therapeutic targets for treating CRC.

## Introduction

Colorectal cancer (CRC) is globally prevalent as the third most frequently diagnosed cancer and stands as the second major contributor to cancer-related deaths, underscoring its status as a critical global health problem 1. Despite advances in early detection, approximately 25% of CRC patients are diagnosed at an advanced stage, and about 25-50% of patients in the early stage have a high risk of developing metastasis 2. Current therapeutic strategies, including surgery and chemotherapy, are often ineffective for patients with advanced CRC, and resistance to chemotherapy is common. Despite recent advances in emerging targeted therapy and immunotherapy, the overall efficacy of these treatments is not satisfactory, and the mortality rate of CRC remains high. Therefore, it is pressing to identify new anti-CRC agents or explore new effective drug targets for CRC therapy.

The PI3K/AKT signaling pathway is a central regulator of cellular processes, including cell growth, proliferation, and survival, and its dysregulation is implicated in cancer initiation and progression 3. Studies have shown that the hyperactivity of the PI3K/AKT pathway is linked to the enhanced proliferation of tumor cells, inhibition of apoptosis, and promotion of epithelial-mesenchymal transition (EMT) 4, 5. Therefore, targeting the PI3K/AKT pathway can be an effective strategy to inhibit tumor progression.

The Warburg effect, characterized by the preferential uptake of glucose and production of lactate by tumor cells even in the presence of oxygen, is a hallmark of cancer metabolism that can support the nutritional and energy demands of tumor cells under adverse conditions 6. Studies have shown that the characterized metabolic features in the tumor microenvironment, such as starvation, hypoxia, and high acidity, may cause precancerous cells to select for the Warburg effect phenotype through transcriptional reprogramming 7.

There is an emerging link between the Warburg effect and the PI3K/AKT signaling pathway. For example, the glycolytic enzyme lactate dehydrogenase A (LDHA) can be induced by the PI3K/AKT signaling pathway, and the ablation of LDHA diminishes the phosphorylation of AKT and the activity of the PI3K/AKT pathway 8. In addition, STYK1, a serine/threonine/tyrosine kinase 1, plays a crucial part in the regulation of glycolysis and the promotion of the Warburg effect via activating the PI3K/AKT pathway 9. Moreover, the metastasis-associated in colon cancer 1 (MACC1) protein, which is associated with tumor invasion and metastasis, has been implicated in promoting the Warburg effect via the PI3K/AKT pathway in gastric cancer 10. Given the importance of the PI3K/AKT pathway and the Warburg effect in tumor cell proliferation, targeting both simultaneously may hold great promise for CRC therapy.

Chloroquine (CQ), a widely used antimalarial drug, which is also included in the World Health Organization Standard List of Essential Medicines, has also been mentioned as a potential agent for combating cancer. It has been shown to inhibit autophagy and has been used as an adjunct for cancer treatment, such as in combination with cisplatin, pterostilbene, and gemcitabine 11-13. In addition, CQ can also induce lysosomal and mitochondrial membrane permeability, trigger the caspase cascade, and induce apoptosis in cancer cells, independent of its autophagy inhibitory effect 14, 15. While the anticancer effect of CQ in CRC has been reported, the underlying mechanisms beyond its known interaction with palmitoyl-protein thioesterase 1 (PPT1), a lysosomal protein highly expressed in multiple cancer cells and involved in the removal of long-chain fatty acids from specific proteins 16, remain largely unexplored.

In this study, using a systematic target identification strategy based on the cellular thermal shift assay (CETSA) 17, we found that CQ directly targets choline kinase alpha (CHKA), a key enzyme in the choline metabolic pathway, and ATP-dependent 6-phosphofructokinase, muscle type (PFKM), a key rate-limiting enzyme in the glycolytic pathway. Binding of CQ to CHKA and PFKM results in the inhibition of their expression and enzymatic activity. CHKA, known to be overexpressed in a variety of cancers and associated with the PI3K/AKT signaling pathway 18, 19, was found to be downregulated after CQ treatment, leading to a significant reduction in PI3K and AKT phosphorylation and tumor cell proliferation. Furthermore, CHKA downregulation also decreases the PFKM expression and inhibits glycolysis. These results suggest a dual role for CQ in inhibiting CHKA and PFKM, thereby suppressing the PI3K/AKT pathway and the Warburg effect, leading to its anti-CRC effects.

## Results

### CQ inhibits CRC cell proliferation and induces apoptosis

**Figure 1:**
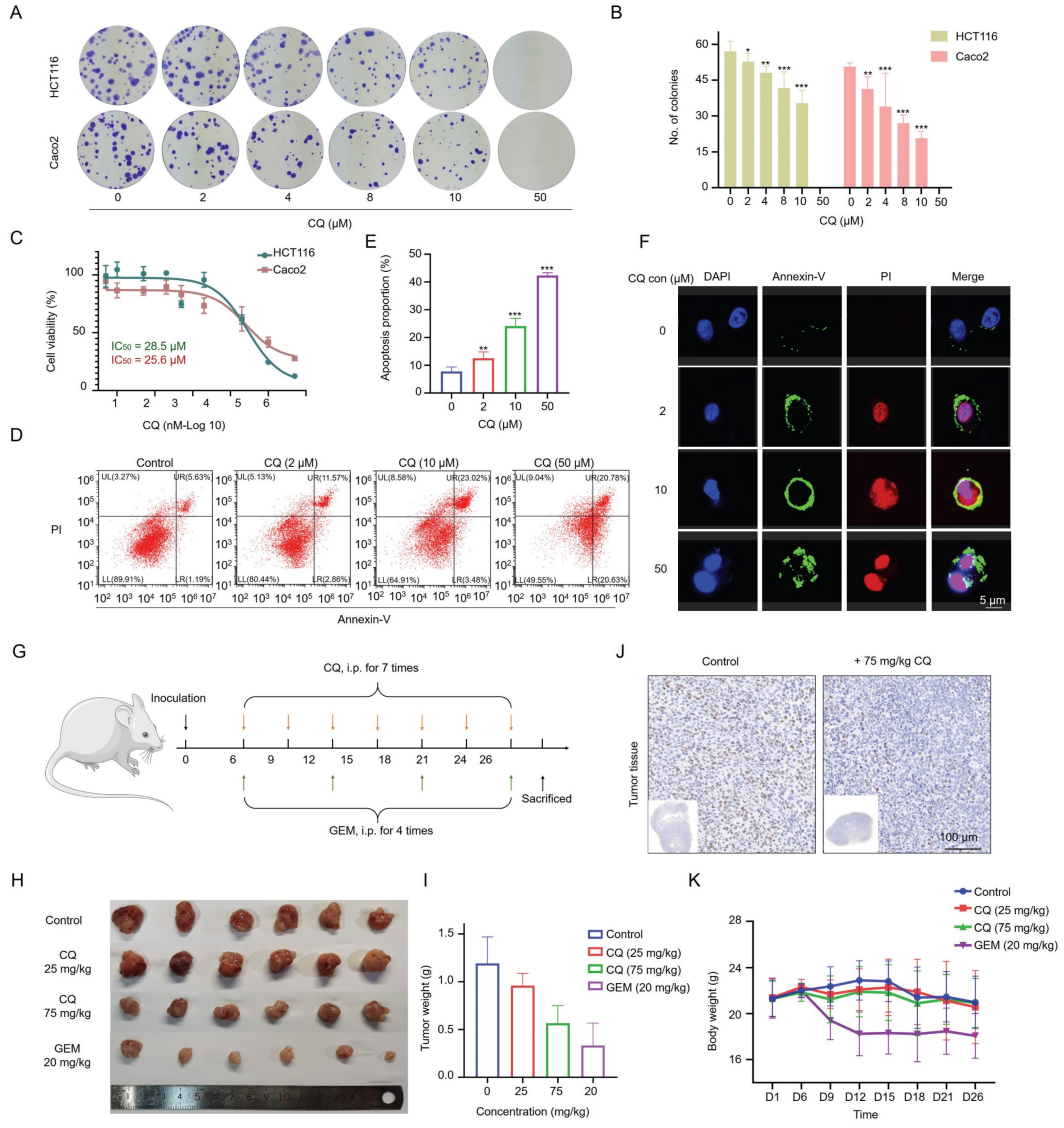
CQ suppresses the proliferation of colorectal cancer (CRC) cells *in vitro* and *in vivo*. **A**. Colony formation of CRC cells after incubation with different concentrations of CQ. **B**. Number of colony formation of HCT116 and Caco2 cells, respectively, after treatment with different doses of CQ (n = 3). **C**. Cytotoxicity of CQ in HCT116 and Caco2 cells after treatment with different CQ concentrations for 48 hours. **D**. Flow cytometry analyses of CQ-induced apoptosis in Caco2 cells. **E**. Proportions of apoptotic Caco2 cells after treatment with different CQ concentrations. **F**. Confocal fluorescence microscope to detect the apoptosis induction of CQ in Caco2 cells after staining with Annexin V/PI. **G**. Schematic diagram of the tumor model in mice being treated with different drugs. **H, I**. Tumor volume (**H**) and weight (**I**) changes in each group after treatment with different concentrations of CQ or the positive drug (Gemcitabine, GEM, 20 mg/kg) (mean ± SD, n = 6). **J**. Photomicrographs of H&E staining of tumor tissues (scale bar, 100 µm). **K**. Body weight changes in each group during the animal experiment (mean ± SD, n = 6). *P < 0.05, **P < 0.01, ***P < 0.001.

While there have been reports on the potential use of CQ for treating CRC, its specific targets and underlying mechanisms in CRC have not been fully elucidated. We first evaluated the anti-proliferative efficacy of CQ in the CRC cell lines HCT116 and Caco2. The colony formation assay confirmed the suppressive impact of CQ on the colony formation of CRC cells (Fig. 1A, B & Fig. S1). The IC_50_ values for CQ treatment at 48 hours in HCT116 and Caco2 cells were 28.5 μM and 25.6 μM, respectively, indicating the suppression of CRC cell viability by CQ (Fig. 1C). Out of these two cell lines, Caco2 cells were slightly more sensitive to CQ, so we selected Caco2 cells for the following study. The effect of CQ on the induction of cell apoptosis was assessed by Annexin-V/PI staining through flow cytometry and confocal fluorescence imaging, showing a dose-dependent increase of the apoptotic cells after CQ treatment (Fig. 1D, E, F). These results suggested that CQ could effectively inhibit the proliferation of CRC cells and induce apoptosis.

### CQ inhibits the growth of CRC xenograft tumors *in vivo*

Next, we evaluated the effect of CQ on the growth of CRC xenograft tumors* in vivo*. Caco2 cells were subcutaneously inoculated into mice, and xenograft tumor growth was monitored following the administration of CQ or gemcitabine (GEM) (Fig. 1G). Tumor sizes were significantly reduced after CQ administration, especially in the high-dose (75 mg/kg) group, without affecting the body weight of tumor-bearing mice; whereas the GEM treatment reduced both tumor size and body weight (Fig. 1H, I and K). The immunohistochemical staining results of Ki67, a nuclear antigen linked to cell proliferation, indicated markedly suppressed tumor cell proliferation upon CQ treatment (Fig. 1J), which were consistent with the *in vitro* results (Fig. 1A, B).

### Identification of CQ targets via cellular thermal shift assay

**Figure 2:**
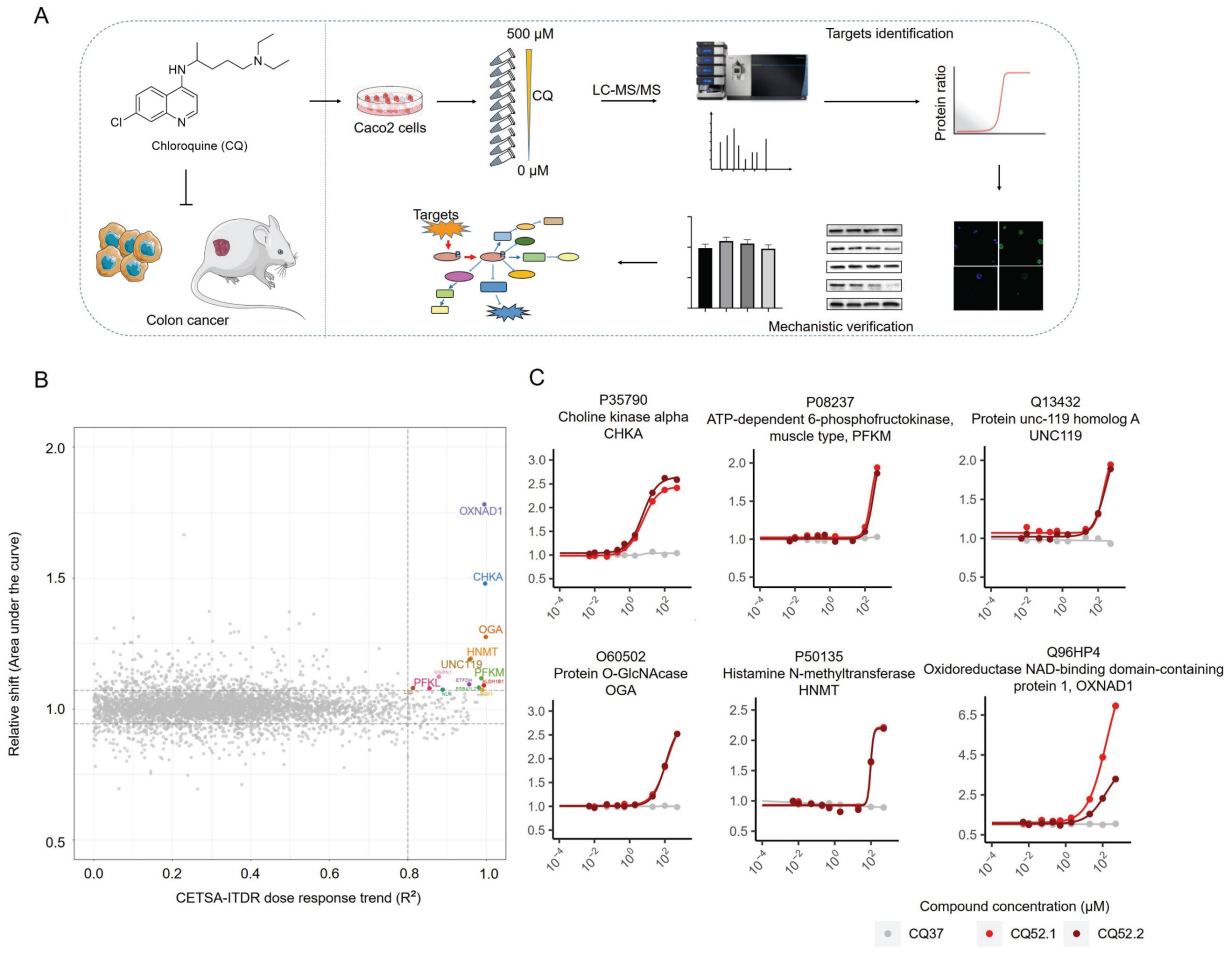
Mass spectrometry-coupled Cellular Thermal Shift Assay (MS-CETSA) identifies the direct binding targets of CQ in Caco2 cells. **A**. Flow chart of experimental design for target identification and mechanistic verification. **B**. An R^2^-AUC plot shows the protein thermal stability shift of the Caco2 cell proteome after incubation with different concentrations of CQ. The potential targets are highlighted in colors other than gray and the gene names are labelled. **C**. Thermal shift profile of the six potential protein targets.

To identify the direct binding targets of CQ against CRC, we employed the mass spectrometry-coupled cellular thermal shift assay (MS-CETSA), a recently developed proteome-wide, label-free target identification method 20-23. After dosing different concentrations of CQ into Caco2 lysates and heating the samples at 37°C or 52°C, changes in protein thermostability across the whole proteome were analyzed by quantitative proteomics (Fig. 2A). Among the 4527 human proteins measured, 14 proteins showed significant positive changes in thermal stability in response to CQ, and these were identified as candidate interacting proteins (Fig. 2B & Table S1). Among these, choline kinase alpha (CHKA, P35790) and ATP-dependent 6-phosphofructokinase, muscle type (PFKM, P08237) caught our attention (Fig. 2C). CHKA, a key enzyme in the first step of phosphatidylcholine synthesis 24, is upregulated across a diverse array of tumors and correlates with the prognosis of cancer patients 25, 26. PFKM, a rate-limiting enzyme in the glycolytic pathway, directly affects the Warburg effect in tumor cells. Therefore, these two proteins were selected for further investigation.

### CQ directly targets CHKA and PFKM and inhibits the Warburg effect

**Figure 3:**
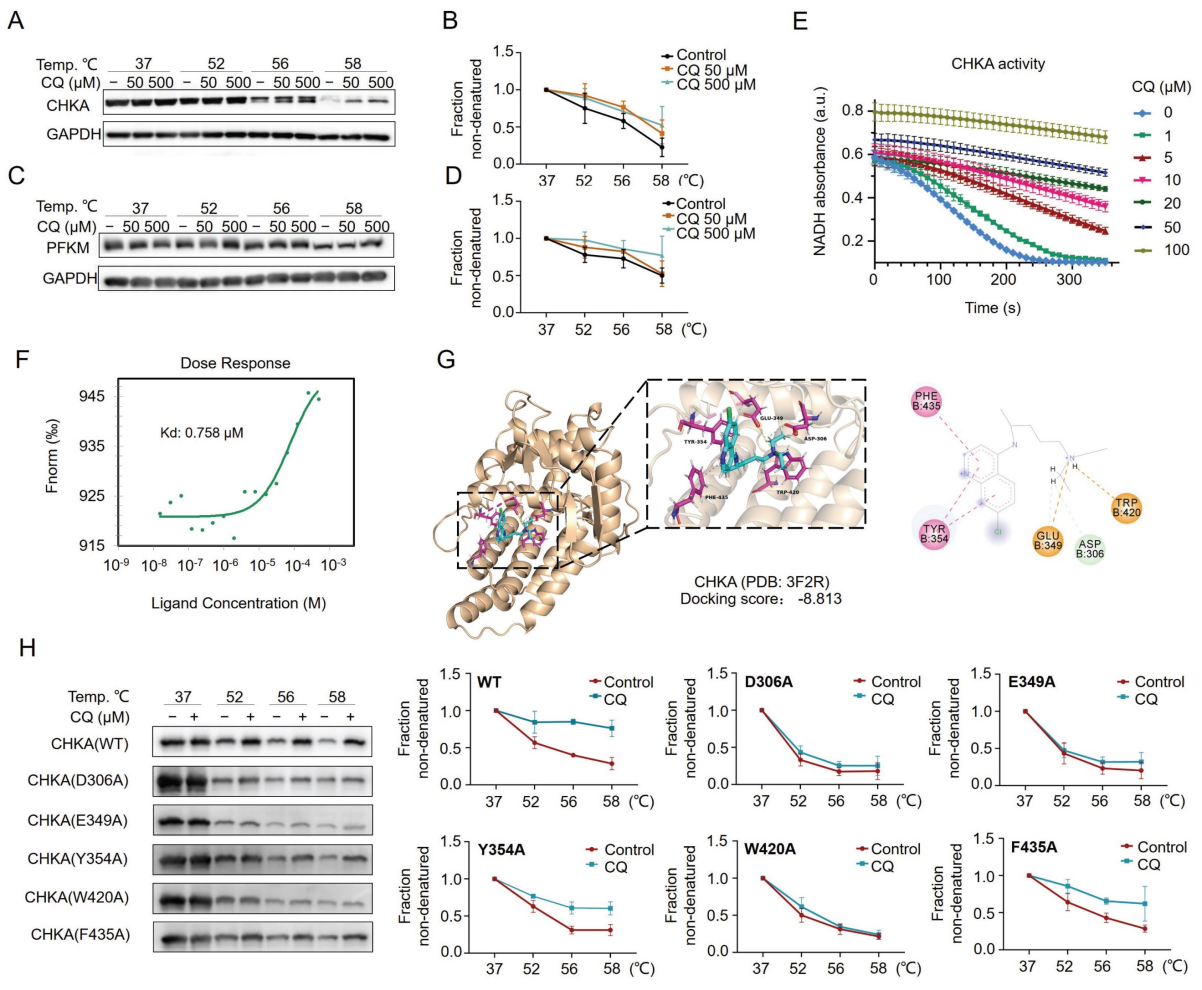
CQ directly targets CHKA and PFKM and inhibits CHKA's catalytic activity. **A-D**. Direct interactions between CQ and CHKA or PFKM are verified by CETSA-Western blot analysis (n = 3). **E**. Decrease of CHKA's activity after treatment with CQ (n = 3). **F**. Measurement of binding affinity between CQ and CHKA by microscale thermophoresis (MST) technique. **G**. Molecular docking and the identification of potential binding sites of CQ and CHKA. **H**. CETSA-Western blot analysis of the binding between CQ and wide type (WT) CHKA or its mutants (n = 3).

CETSA coupled with Western blot (CETSA-WB) was performed to verify the direct interaction between CQ and CHKA or PFKM. The results showed the increased stability of CHKA and PFKM proteins with increasing concentrations of CQ (Fig. 3A-D). We then investigated the inhibition of CHKA kinase activity by CQ. Notably, the activity of CHKA was suppressed by the increasing concentration of CQ (Fig. 3E). Next, we expressed and purified the recombinant CHKA protein and measured its binding affinity for CQ using the microscale thermophoresis (MST) technique. The results reflected a binding between CHKA and CQ, with a dissociation constant (*K*_D_) of 0.758 μM (Fig. 3F). Molecular docking suggested that CQ could interact with the D306, E349, Y354, W420, and F435 sites on the CHKA protein (Fig. 3G & Fig. S2). We therefore constructed five CHKA mutants in which each of these five sites was mutated to alanine (A), respectively. The interaction between CQ and CHKA mutants was assessed using CETSA-WB. For the D306A, E349A, and W420A mutants, CQ treatment failed to increase the protein thermostability (Fig. 3H). The extent of stability increase in Y354A and F435A mutants was much smaller compared to the WT CHKA protein (Fig. 3H). These results indicated that D306, E349, and W420 were likely to be the direct binding sites of CHKA for CQ.

**Figure 4:**
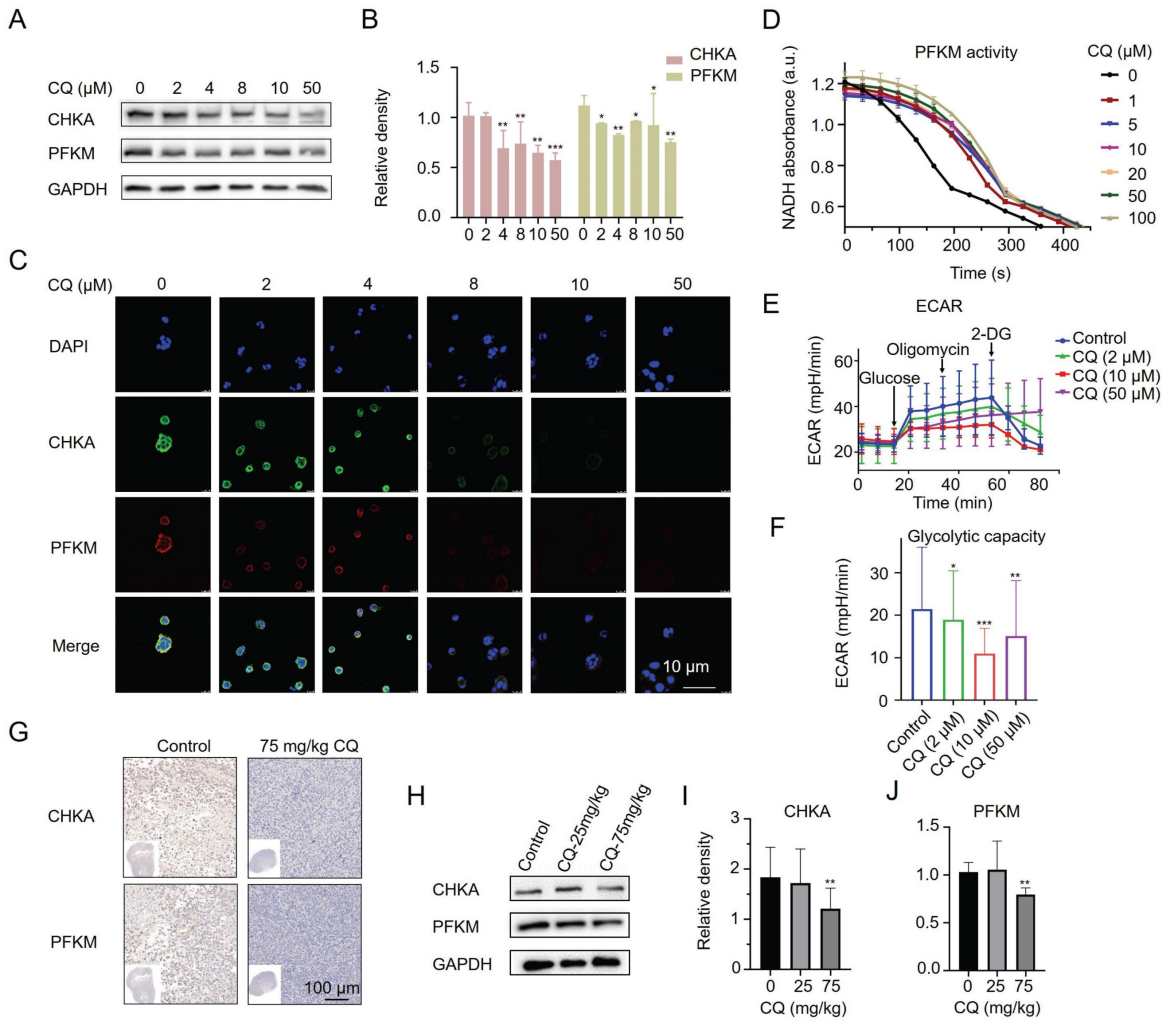
CQ decreases the protein levels of CHKA and PFKM *in vitro* and *in vivo*. **A, B**. Western blot analysis of the level changes of CHKA and PFKM proteins in Caco2 cells after treatment with different concentrations of CQ (n = 3). **C**. Confocal fluorescence images of CHKA and PFKM proteins in Caco2 cells after incubation with different concentrations of CQ (Scale bar, 10 µm). **D**. Measurement of cellular PFKM activity in Caco2 cells after treatment with CQ (n = 3). **E, F**. ECAR (**E**) and glycolysis stress test (**F**) of Caco2 cells after treatment with CQ (n = 3). **G**. Immunohistochemical staining of tumor tissues with CHKA and PFKM monoclonal antibodies, respectively (scale bar, 100 µm). **H-J**. Western blot analysis of the level changes of CHKA and PFKM proteins in tumor tissues after treatment with different doses of CQ (n = 3). *P < 0.05, **P < 0.01, ***P < 0.001.

Furthermore, we found that CQ treatment decreased the protein levels of CHKA and PFKM in Caco2 cells by Western blot (Fig. 4A, B) and confocal fluorescence imaging (Fig. 4C). We also examined whether the activity of cellular PFKM was affected by CQ. As expected, PFKM activity was dramatically blocked even after treatment with as low as 1 μM CQ (Fig. 4D). Given the important role of PFKM in glycolysis, we next evaluated the effect of CQ on glycolysis. CQ treatment decreased the extracellular acidification rate (ECAR) of Caco2 cells in a concentration-dependent manner, suggesting that CQ could block the Warburg effect in tumor cells (Fig. 4E, F). Surprisingly, Caco2 cells treated with a high concentration of CQ (50 μM) became insensitive to 2-DG, a hexokinase inhibitor, suggesting that CQ may directly or indirectly influence the expression or activity of hexokinase. In addition, immunohistochemical staining and Western blot analysis further confirmed the inhibitory effect of CQ on the expression of CHKA and PFKM proteins in tumor tissues (Fig. 4G-J & Fig. S3).

### CQ inhibits the PI3K/AKT signaling pathway and suppresses tumor cell growth

**Figure 5:**
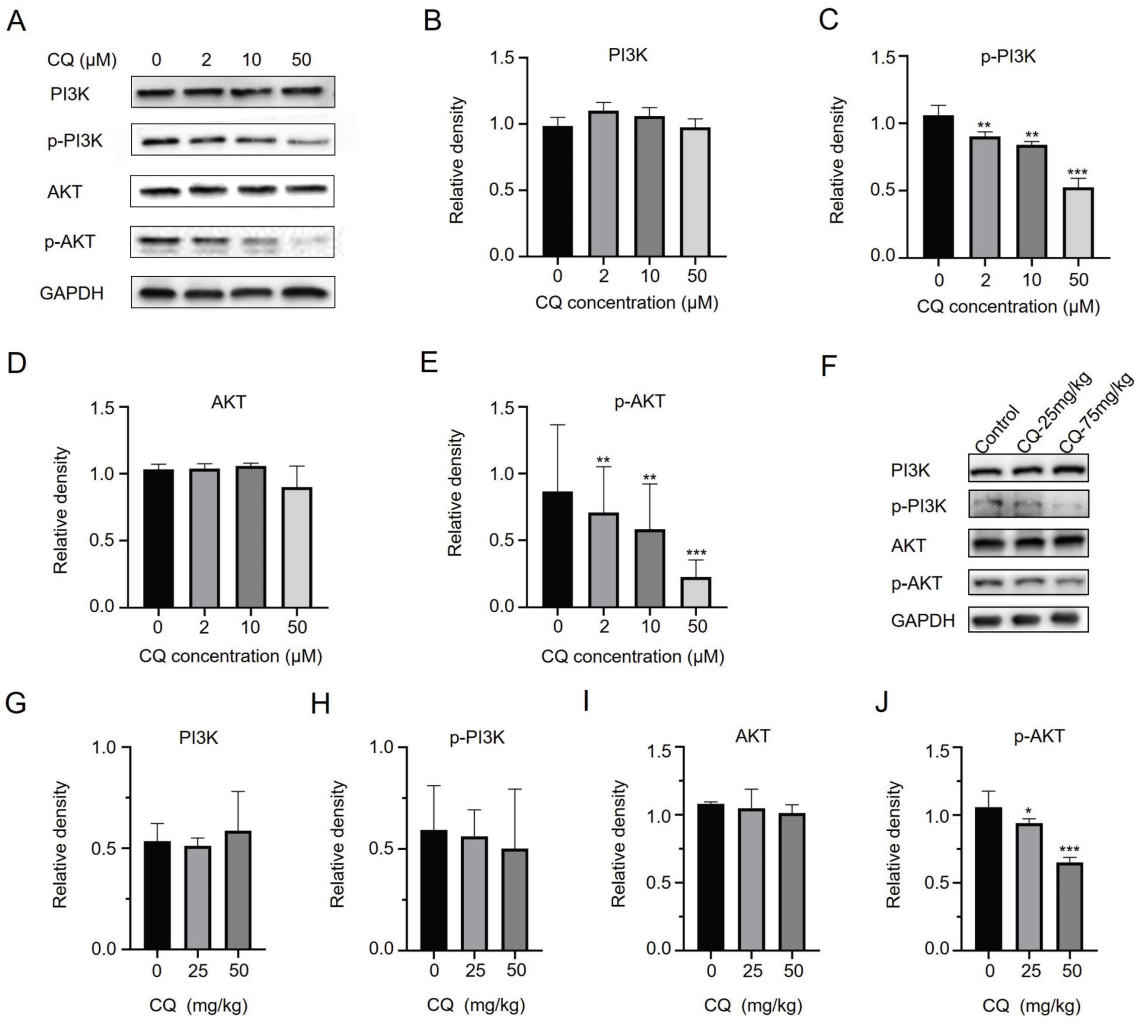
CQ suppresses the growth of CRC cells by inhibiting the PI3K/AKT signaling pathway. **A-E**. Western blot analysis of the level changes of key proteins in the PI3K/AKT signaling pathway after treatment with different concentrations of CQ *in vitro* (n = 3). **F-J**. Western blot analysis of the level changes of key proteins in the PI3K/AKT signaling pathway after treatment with different doses of CQ *in vivo* (n = 3). *P < 0.05, **P < 0.01, ***P < 0.001.

The above results suggested that CQ directly binds to CHKA and PFKM, inhibiting their expressions and enzyme activities. As the expression and activity of CHKA are closely related to the PI3K/AKT signaling pathway 18, 19, we next sought to detect changes in the expression and phosphorylation level of PI3K and AKT. CQ treatment resulted in decreased phosphorylation levels of PI3K and AKT without altering their expressions *in vitro* (Fig. 5A-E) and *in vivo* (Fig. 5F-J & Fig. S4). The decrease in phosphorylation of PI3K and AKT was consistent with the reduction of CHKA levels (Fig. 4A). These data indicated that CQ suppressed the PI3K/AKT pathway, thereby inhibiting tumor growth and proliferation.

### CHKA downregulation impairs the PI3K/AKT signaling and inhibits cell proliferation

**Figure 6:**
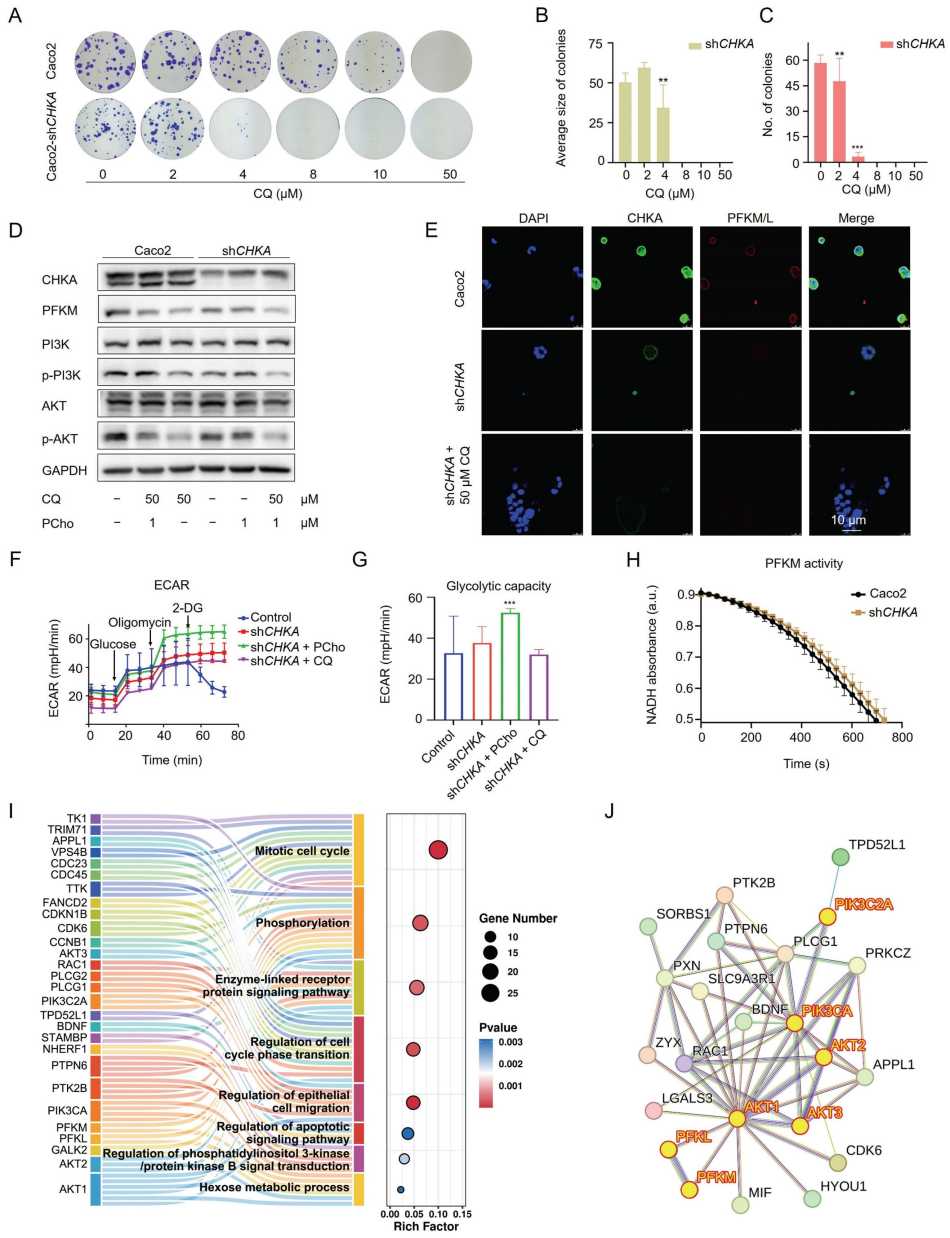
Downregulation of CHKA impairs the PI3K/AKT signaling pathway and restrains the Warburg effect in Caco2 cells. **A**. Colony formation assay of Caco2 and Caco2-sh*CHKA* cells after treatment with different concentrations of CQ. **B, C**. Statistical analysis of the size and number of colonies in Caco2-sh*CHKA* cells after treatment with different concentrations of CQ (n = 3). **D**. Western blot analysis of the level changes of CHKA, PFKM, and key proteins in the PI3K/AKT signaling pathway between Caco2 and Caco2-sh*CHKA* cells after CQ treatment. PCho, phosphorylcholine. **E**. Confocal fluorescence images of CHKA and PFKM in Caco2 and Caco2-sh*CHKA* cells after CQ treatment (Scale bar, 10 µm). **F, G**. Glycolysis stress test of Caco2-sh*CHKA* cells after treatment with CQ or PCho (n = 3). **H**. Measurement of PFKM activity changes after CHKA knockdown (n = 3). **I**. GO pathways enriched by all DEPs. **J**. Protein-protein interaction (PPI) analysis of DEPs involved in phosphorylation regulation, enzyme-linked receptor protein regulation, apoptosis, and hexose metabolic process.

To further establish the relationship between CHKA and the PI3K/AKT signaling pathway, we first knocked down CHKA levels in Caco2 cells using shRNA. Among the four designed shRNAs, shRNA3 showed the best knockdown effect (Fig. S5). Therefore, it was selected for downstream experiments. CHKA knockdown significantly reduced the dimension and quantity of colonies in the colony formation assay (Fig. 6A-C), indicating that CHKA is important for the proliferation of CRC cells. CHKA knockdown also significantly reduced the phosphorylation levels of PI3K and AKT, which was reversed by the addition of phosphorylcholine (PCho), a downstream product catalyzed by CHKA (Fig. 6D). These results demonstrated that CHKA was indeed related to the PI3K/AKT signaling pathway and that CQ inhibited tumor cell proliferation by directly targeting CHKA and blocking the PI3K/AKT signaling pathway.

### Inhibition of the PI3K/AKT signaling affects the Warburg effect

Knockdown of CHKA in Caco2 cells not only suppressed the PI3K/AKT pathway but also reduced the expression of PFKM (Fig. 6D, E), which was reversed by CQ deprivation or PCho addition. This suggests that CHKA downregulation and PI3K/AKT inhibition may attenuate the Warburg effect. We then compared the glycolytic capacity of Caco2 cells before and after CHKA knockdown. The results indicated that CHKA downregulation decreased the ECAR of Caco2 cells, and the addition of PCho significantly provided anaplerosis for the glycolysis in Caco2-sh*CHKA* cells, suggesting that inhibition of the PI3K/AKT pathway could efficiently block the Warburg effect in tumor cells (Fig. 6F, G). Unexpectedly, Caco2-sh*CHKA* cells were more sensitive to oligomycin than Caco2, indicating that CHKA knockdown increased the cell sensitivity to oligomycin (Fig. 6F). However, Caco2-sh*CHKA* cells were insensitive to 2-DG (Fig. 6F), which was consistent with the results of 50 μM CQ treatment in Caco2 cells (Fig. 4E, F), suggesting that CHKA may directly or indirectly affect the expression or activity of hexokinase. In addition, PFKM activity was slightly decreased in Caco2-sh*CHKA* cells (Fig. 6H). These data showed that CHKA downregulation reduced the expression of PFKM and restrained its activity, thereby blocking the Warburg effect.

Subsequently, we investigated the differentially expressed proteins (DEPs) and their associated signaling pathways in Caco2-sh*CHKA* cells using LC-MS/MS. GO enrichment analysis revealed that the 264 DEPs primarily participated in phosphorylation regulation, cell cycle, enzyme-linked receptor protein regulation, epithelial cell migration, apoptosis, regulation of phosphatidylinositol 3-kinase/protein kinase B signal transduction and hexose metabolic process, further indicating the close relationship of CHKA to the PI3K/AKT pathway and the Warburg effect (Fig. 6I). Next, we performed a protein-protein interaction (PPI) network analysis for the DEPs that are involved in the phosphorylation, enzyme-linked receptor protein regulation, apoptosis, and hexose metabolic process, as the targets in this study were mainly related to these pathways. The results suggested that AKT, PIK3C, PFKM, and PFKL are highly connected (Fig. 6J). These results also supported that CQ exerted its anti-CRC effect by inhibiting the PI3K/AKT pathway and the Warburg effect.

## Discussion

In this study, we explored the potential of CQ in treating CRC. We showed that CQ effectively inhibited the proliferation of CRC cells both *in vitro* and *in vivo*. Through target identification using MS-CETSA, we found that CHKA and PFKM were the direct binding targets of CQ in Caco2 cells, and these targets were located in the cytoplasm. It's worth noting that before our study, the only confirmed direct protein of CQ, a well-known lysosomotropic drug, was PPT1, a target protein mainly found in lysosomes 16. CHKA and PFKM are rate-limiting enzymes in the choline metabolic and glycolytic pathways, respectively, and their suppression of expression and enzyme activity after CQ treatment is associated with decreased phosphorylation of PI3K and AKT, as well as attenuated the Warburg effect. The PI3K/AKT signaling pathway and the Warburg effect are essential pathways for tumor initiation and progression.

Our results revealed that the PI3K/AKT signaling pathway was related to CHKA in which the knockdown of CHKA also reduced the phosphorylation levels of PI3K and AKT, and these effects were rescued after the addition of PCho, a downstream product catalyzed by CHKA, both in Caco2 cells treated with CQ and in Caco2-sh*CHKA* cells. These findings are consistent with the emerging relationship between the PI3K/AKT signaling pathway and the Warburg effect 27. How does the knockdown of CHKA affect the PI3K/AKT pathway? One possible explanation is the knockdown of CHKA lowered the intracellular level of PCho, a necessary precursor for the synthesis of several lipid second messengers, including lyso-phosphatidic acid, diacylglycerol, lyso-phosphatidylcholine and phosphatidic acid that are capable of activating oncogenic signaling 28, 29. Yalcin *et al.* demonstrated that phosphatidic acid is a key activator of the mitogen-activated protein kinase (MAPK) and PI3K/AKT signaling pathways 30. CHKA downregulation also decreased the expression and activity of PFKM *in vitro*, which may be attributed to the knockdown of CHKA reducing the phosphorylation levels of PI3K and AKT, subsequently leading to the downregulation of PFKM. This is consistent with the results observed in the study of Jeon *et al.* 31. Therefore, the capacity of CQ in dual inhibition of CHKA and PFKM results in the simultaneous suppression of the PI3K/AKT pathway and the Warburg effect.

We also performed experiments to assess the tumorigenic capacity of Caco2-sh*CHKA* cells. We injected approximately 1 × 10^7^ Caco2-sh*CHKA* or Caco2 cells subcutaneously into the right axilla of BALB/c nude mice. In the Caco2-sh*CHKA* group, the tumor could reach a size of about 6 mm^3^ two weeks after cell inoculation, which was much smaller than that of the Caco2 tumor group inoculated at the same time, but the Caco2-sh*CHKA* tumor tended to stop growing after reaching about 50 mm^3^, followed by shrinkage and eventual disappearance (data not shown); we were therefore unable to establish a tumor xenograft model for Caco2-sh*CHKA* cells. This experiment was performed three times and failed each time. Nevertheless, these results suggest that CHKA is essential for tumor growth.

We further compared the glycolytic function of Caco2 and Caco2-sh*CHKA* cells and revealed that CHKA knockdown impaired the glycolytic capacity of tumor cells. Notably, Caco2-sh*CHKA* cells were more sensitive to oligomycin than Caco2 cells. In contrast, Caco2-sh*CHKA* cells became insensitive to 2-DG, an inhibitor of hexokinase in glycolysis, consistent with the result of 50 μM CQ treatment in Caco2 cells. Oligomycin is an inhibitor of the proton-pumping activity of mitochondrial ATP synthase and suppresses mitochondrial ATP synthesis 32. Our data suggest that CHKA may be related to mitochondrial function and may directly or indirectly affect the expression or activity of hexokinase in glycolysis, which is worthy of further investigation. These results confirm that the Warburg effect has a strong correlation with the CHKA/PI3K/AKT signaling pathway.

**Figure 7:**
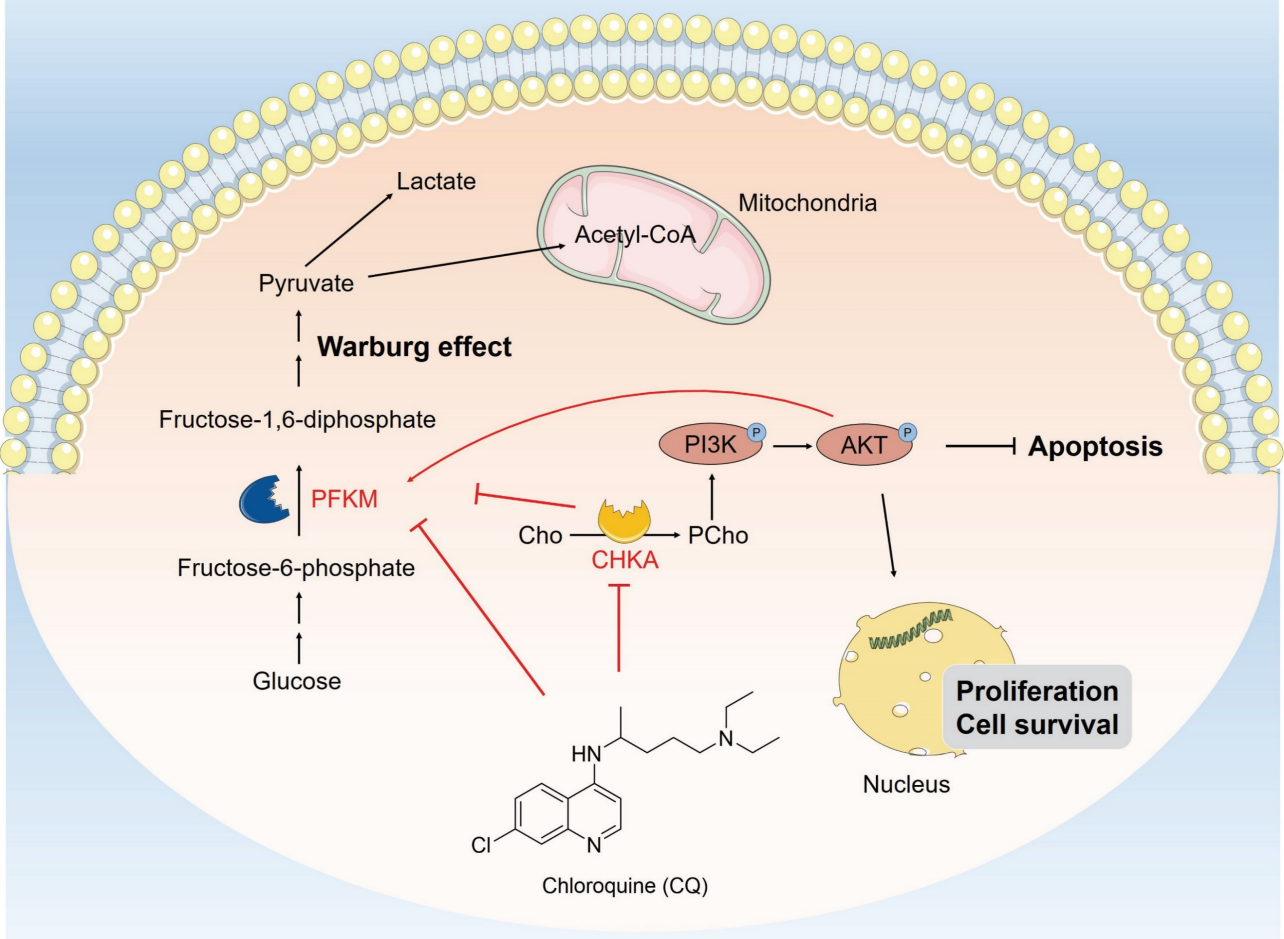
Schematic description of CQ suppresses cell proliferation and induces apoptosis through inhibiting the PI3K/AKT signaling pathway and the Warburg effect in colorectal cancer. CQ specifically binds to CHKA and PFKM, inhibiting their expressions and enzyme activities and restricting the phosphorylation of PI3K and AKT, thereby inhibiting the PI3K/AKT pathway and the Warburg effect, exerting its anti-CRC effect.

In conclusion, our study reveals that CQ exerts its antitumor effects by directly binding to CHKA and PFKM, inhibiting their expression and enzyme activity, thereby blocking the PI3K/AKT signaling pathway and the Warburg effect (Fig. 7). Our findings not only enhance comprehension regarding the antitumor mechanisms of CQ, but also provide valuable therapeutic targets for the treatment of CRC.

## Materials and Methods

### Cell culture

The human colorectal adenocarcinoma epithelial cell lines, Caco2 and HCT116, were employed in this study. Cells were propagated in MEM medium (Corning) supplemented with 20% fetal bovine serum (FBS), 1% non-essential amino acids (NEAA), and 1% penicillin/streptomycin. Cultivation was conducted at 37°C in a humidified incubator containing 5% CO_2_.

### Cell viability assay

Cells were exposed to a range of CQ concentrations (0 ~ 500 μM), with each concentration replicated across four wells. CCK-8 kit was used to evaluate the cell viability 48 hours after treatment, and measured using a 96-well plate reader (EnVision2105, PerkinElmer, USA).

### Colony formation

Caco2 cells were plated in a 6-well plate at a density of 3 × 10^2^ cells per well and incubated with different concentrations of CQ (0, 2, 4, 8, 10, 50 μM) at 37°C for two weeks. Following a 30-minutes staining with 0.1% crystal violet, the colonies were visualized and counted.

### Flow cytometry

For flow cytometry analysis, Caco2 cells that treated with different concentrations of CQ were stained with Annexin V and propidium iodide (PI) in serum-free MEM. Cells were harvested and single-cell suspensions were prepared and quantified using a FACSort Flow Cytometer (Beckman Coulter, Brea, CA, USA) at 488 nm. To exclude cellular debris, an appropriate threshold based on forward light scatter was employed.

### Western blot

In the Western blot analysis, proteins were separated using SDS-PAGE gel and then transferred onto PVDF membranes (Millipore) and blocked with a solution containing 5% bovine serum albumin (BSA). Subsequently, the membranes were incubated separately with primary and secondary antibodies (as shown in Table S2). Protein bands were visualized by employing Tanon^TM^ High-sig ECL Substrate (Tanon, China).

### Immunofluorescence staining and confocal microscopy

Cells were cultured in poly-lysine-coated Petri dishes and fixed with fresh para-formaldehyde (4%), followed by washing with precooled PBS 3 times. Then, cells were treated with (0.1 ~ 0.25%) Triton X-100 to improve antibody permeability. Cells were incubated with TBST containing 5% BSA before incubated with anti-CHKA antibody. Cells were consecutively incubated with a secondary fluorescence antibody. Next, cells were incubated with anti-PFKM/L antibody. Finally, cells were stained with Hoechst, closed with PBS and visualized using a confocal microscope (Leica TCS SP8 SR).

### Generation of *CHKA* knockdown stable cell line

The pLKO.1 vector was used to construct the sh*CHKA* plasmid. The sh*CHKA* oligos (Forward oligo: CCGGGCCAGATATTTCTGCAGAAATCTCGAGATTTCTGCAGAAATATCTGGCTTTTTG; Reverse oligo: AATTCAAAAAGCCAGATATTTCTGCAGAAATCTCGAGATTTCTGCAGAAATATCTGGC) were inserted into the AgeI and EcoRI sites of the pLKO.1 vector. For the generation of lentiviral particles, HEK293T cells were transfected with a mixture of pLKO.1 sh*CHKA* plasmid, psPAX2 packaging plasmid, and pMD2.G envelope plasmid at a ratio of 4:3:1 using lipofectamine2000 transfection reagent (Thermo Scientific), and cells were incubated at 37°C, 5% CO_2_ for 15 hours. The transfection media was replaced with fresh DMEM containing 10% FBS, then lentiviral particles were harvested. For Caco2 cells infection, 500 uL of lentiviral particles were added to a 60 mm culture dish, and fresh media supplemented with 6 ug/ml puromycin (pre-determined optimal concentration) were added 24 hours after infection for selection. An uninfected dish of cells was used in parallel as a positive control for selection.

### Recombinant protein expression and purification

The coding sequences for CHKA, pyruvate kinase (PKM), and lactate dehydrogenase (LDH) were cloned into the pET-28a vector, respectively. Protein purification was performed as previously described 20.

### CHKA activity inhibition assay

Choline kinase activity was measured using a modified PKM/LDH-coupled system 33. The reaction buffer was composed of 100 mM Tris-HCl (pH 7.5), 150 mM KCl, 2 mM choline chloride, 12 mM MgCl_2_, 0.4 mM NADH, 1 mM phosphoenolpyruvate, 5 mM ATP, 2 μg PKM, 2 μg LDH, and 2 μg CHKA in a total volume of 1 mL. The ADP released by the consumption of ATP by CHKA was used as a substrate for phosphoenolpyruvate catalyzed by PKM. The resulting pyruvate can be catalyzed by LDH to produce lactate by consumption of NADH. CHKA activity was followed by continuous monitoring of the decrease in NADH at 340 nm using a 96-well plate reader (EnVision2105, PerkinElmer, USA).

### PFKM activity inhibition assay

A PFK kit (Solarbio, China) was used to detect the PFKM activity. Caco2 lysate containing PFKM was incubated with CQ (0 ~ 100 μM) for 30 min, followed by the addition of detection buffer and continuous monitoring of NADH at 340 nm using a 96-well plate reader (EnVision2105, PerkinElmer, USA).

### Molecular docking

The binding affinity of CQ to CHKA was evaluated using Schrodinger 2018 software. The crystal structure of CHKA with PDB: 3F2R was selected for molecular docking using Discovery Studio 2016, and the Docking Score module was used to evaluate the protein-ligand binding affinity. The 3D images of CQ and CHKA were generated using PyMOL 2.5.

### Verification of binding sites between CQ and CHKA

The amino acids of the five possible binding sites of CQ were mutated to alanine (D306A, E349A, Y354A, W420A, F435A). All mutants were constructed in the pET-28a vector, the lysate of cultivated *E. coli* BL21 cell was divided into two parts and one part was treated with 500 μM CQ. After incubation, each part was divided into four portions and heated at different temperatures (37 °C, 52 °C, 56 °C, 58 °C). These samples were then detected by Western blot.

### Glycolysis stress test

The XF Glycolysis Stress Test Kit (Agilent, USA) was used to measure the glycolysis function in cells. Briefly, 1 × 10^4^ cells/well Caco2 or Caco2-sh*CHKA* cells were plated in the XF microplate, followed by treatment with different concentrations of CQ, and the cellular extracellular acidification rate (ECAR) was detected using a Seahorse extracellular flux analyzer (Seahorse Bioscience, USA). Experiments were conducted according to the guidelines provided by the manufacturer, and data analysis was performed using Seahorse XF Wave software (Agilent Technologies, USA).

### Tumorigenicity assay *in vivo*

Approximately 5 × 10^6^ Caco2 cells were subcutaneously injected into the right axilla of BALB/c nude mouse, male, 6 weeks old. After five days, the mice were randomly divided into four groups of eight mice each: the control group, CQ 25mg/kg/day group, CQ 75mg/kg/day group, and the positive drug (Gemcitabine, GEM) group. Tumor formation was monitored in the BALB/c mice for 26 days. Mice weights were measured weekly to assess the toxicity of the drugs.

### Isothermal dose-response MS-CETSA and target identification

Isothermal dose-response (ITDR) MS-CETSA assay for CQ was performed as previously described to identify the potential protein targets of CQ 21.

### LC-MS/MS data analysis

LC-MS/MS data were analyzed using the methods previously described 21.

### Statistical analysis

Data were presented as mean ± standard (SD) deviation based on a minimum of three replicates. Group comparisons were performed using one-way ANOVA through GraphPad Prism software. A P-value less than 0.05 was deemed to indicate statistical significance.

## Supplementary Material

Supplementary figures.

## Abbreviations

CETSA: cellular thermal shift assay
CHKA: choline kinase alpha
CQ: chloroquine
CRC: colorectal cancer
DEP: differentially expressed protein
ECAR: cellular extracellular acidification rate
EMT: epithelial-mesenchymal transition
FBS: fetal bovine serum
ITDR: isothermal dose-response
LDHA: lactate dehydrogenase A
MACC1: metastasis-associated in colon cancer 1
NEAA: non-essential amino acid
PCho: phosphorylcholine
PFK: ATP-dependent 6-phosphofructokinase
PFKM: ATP-dependent 6-phosphofructokinase, muscle type
PKM: pyruvate kinase
PPI: protein-protein interaction
PPT1: palmitoyl-protein thioesterase 1
