# Supplementary material for: Chloroquine Suppresses Colorectal Cancer Progression via Targeting CHKA and PFKM to inhibit the PI3K/AKT Pathway and the Warburg Effect
Source: Int J Biol Sci. 2025 Jan 27;21(4):1619–31. doi: 10.7150/ijbs.101921 (PMC11844273; doi:10.7150/ijbs.101921)
Supplement: Supplementary file 1 — Supplementary figures. [file ijbsv21p1619s1.pdf]

Supplementary Information

Chloroquine Suppresses Colorectal Cancer Progression via Targeting CHKA and PFKM to inhibit the PI3K/AKT Pathway and the Warburg Effect

Yanqing Liu[1#], Yongping Zhu[1#], Liwei Gu[1#], KeXin Li[2#], Ang Ma[1], Li Liu[1], Yuqing Meng[1], Junzhe Zhang[1], Shengnan Shen[1], Qiaoli Shi[1], Dandan Liu[1], Xinwei Zhang[1], Shujie Zhang[1], Xin Chai[1], Peng Gao[1], Jiale Xing[1], Yaxu Wang[3], Honglin Chen[4], Rui Liu[5], Qingfeng Du[6*], Haitao Liu[7*], Lingyun Dai[8*], Jigang Wang[1,3,4,6,8*]

[1] State Key Laboratory for Quality Ensurance and Sustainable Use of Dao-di Herbs, Artemisinin Research Center, and Institute of Chinese Materia Medica, China Academy of Chinese Medical Sciences, Beijing 100700, China.

[2] Department of Traditional Chinese Medicine, Peking Union Medical College Hospital, Peking Union Medical College, Chinese Academy of Medical Sciences, Beijing 100730, China

[3] Key Laboratory of Computational Chemistry-Based Natural Antitumor Drug Research & Development, Liaoning, Shenyang Pharmaceutical University, Shenyang 110016, China.

[4] State Key Laboratory of Antiviral Drugs, School of Pharmacy, Henan University, Kaifeng 475000, China.

[5] College of Animal Science and Technology, Henan Agricultural University, Zhengzhou 450002, China.

[6] School of Traditional Chinese Medicine and School of Pharmaceutical Sciences, Southern Medical University, Guangzhou 510515, Guangdong, China

[7] State Key Laboratory of Bioactive Substance and Function of Natural Medicines, Institute of Medicinal Plant Development, Chinese Academy of Medical Sciences, Peking Union Medical College, Beijing 100193, China

[8] Department of Critical Care Medicine, Guangdong Provincial Clinical Research Center for Geriatrics, Shenzhen Clinical Research Centre for Geriatrics, Department of Nuclear Medicine, Shenzhen People's Hospital (The First Affiliated Hospital, Southern University of Science and Technology; The Second Clinical Medical College, Jinan University), Shenzhen, Guangdong 518020, China.

[*]Corresponding Authors:

Qingfeng Du, School of Traditional Chinese Medicine and School of Pharmaceutical

Sciences, Southern Medical University, Guangzhou 510515, Guangdong, China
E-mail: dqf1689@smu.edu.cn

Haitao Liu, State Key Laboratory of Bioactive Substance and Function of Natural Medicines, Institute of Medicinal Plant Development, Chinese Academy of Medical Sciences, Peking Union Medical College, Beijing 100193, China
E-mail: htliu@implad.ac.cn

Lingyun Dai, Department of Geriatrics, the Second Clinical Medical College of Jinan University, the First Affiliated Hospital of Southern University of Science and Technology, Shenzhen People's Hospital, Shenzhen 518020, Guangdong, China. E-mail: lingyun.dai@outlook.com;

Jigang Wang, Department of Pharmacological Sciences, Yong Loo Lin School of Medicine, National University of Singapore, Singapore 117600, Singapore; State Key Laboratory for Quality Ensurance and Sustainable Use of Dao-di Herbs, Artemisinin Research Center, and Institute of Chinese Materia Medica, China Academy of Chinese Medical Sciences, Beijing 100700, China. E-mail: jgwang@icmm.ac.cn.

[#] These authors contributed equally to this work.

1. Supplementary Tables

Table S1 Potential target proteins of CQ identified by MS-CETSA

No.	Accession	Protein Name	Description
1	O60291	MGRN1	E3 ubiquitin-protein ligase MGRN1
2	P35790	CHKA	Choline kinase alpha
3	Q96HP4	OXNAD1	Oxidoreductase NAD-binding domain-containing protein 1
4	P50135	HNMT	Histamine N-methyltransferase
5	O60502	OGA	Protein O-GlcNAcase
6	Q9BYT8	NLN	Neurolysin, mitochondrial
7	Q13432	UNC119	Protein unc-119 homolog A
8	Q16134	ETFDH	Electron transfer flavoprotein-ubiquinone oxidoreductase, mitochondrial
9	P48449	LSS	Lanosterol synthase
10	P30837	ALDH1B1	Aldehyde dehydrogenase X, mitochondrial
11	P00558	PGK1	Phosphoglycerate kinase 1
12	P17858	PFKL	ATP-dependent 6-phosphofructokinase, liver type
13	P08237	PFKM	ATP-dependent 6-phosphofructokinase, muscle type
14	O43491	EPB41L2	Band 4.1-like protein 2

Table S2 Antibodies used in the manuscript

Product	Supplier	Catatlog No.
PFKM Polyclonal antibody	Proteintech	55028-1-AP
Choline kinase alpha polyclonal antibody	Proteintech	13520-1-AP
PI3K/AKT signaling pathway panel	Abcam	ab283852
GAPDH Monoclonal antibody	Proteintech	60004-1-Ig

2. Supplementary Figures

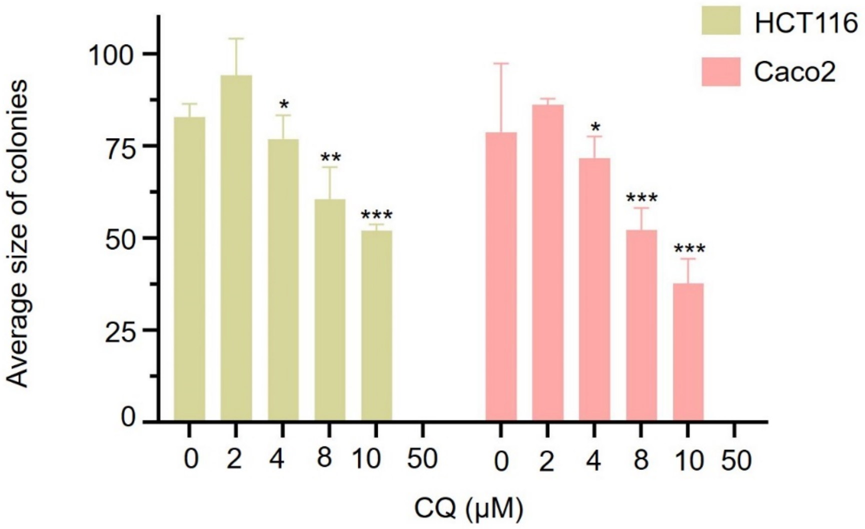

Figure S1. Average size of colony formation of HCT116 and Caco2 cells, respectively, after treatment with different doses of CQ (n = 3).

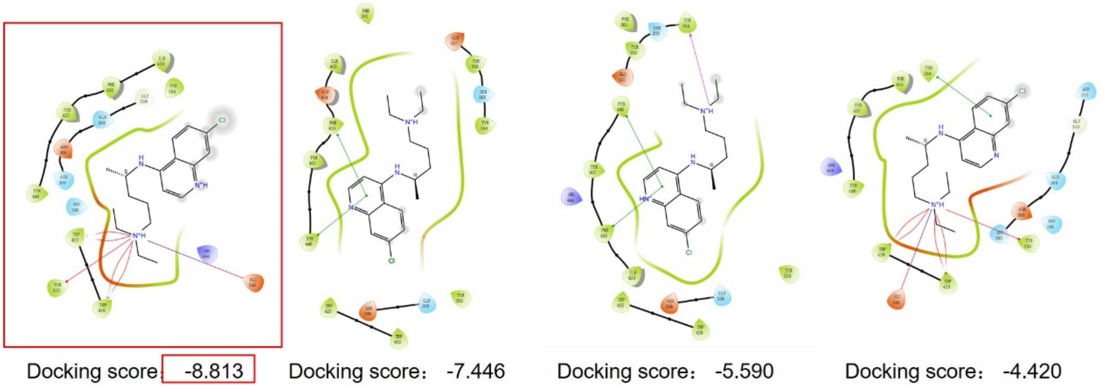

Figure S2. Molecular docking of CQ and CHKA yielded four potential binding modes.

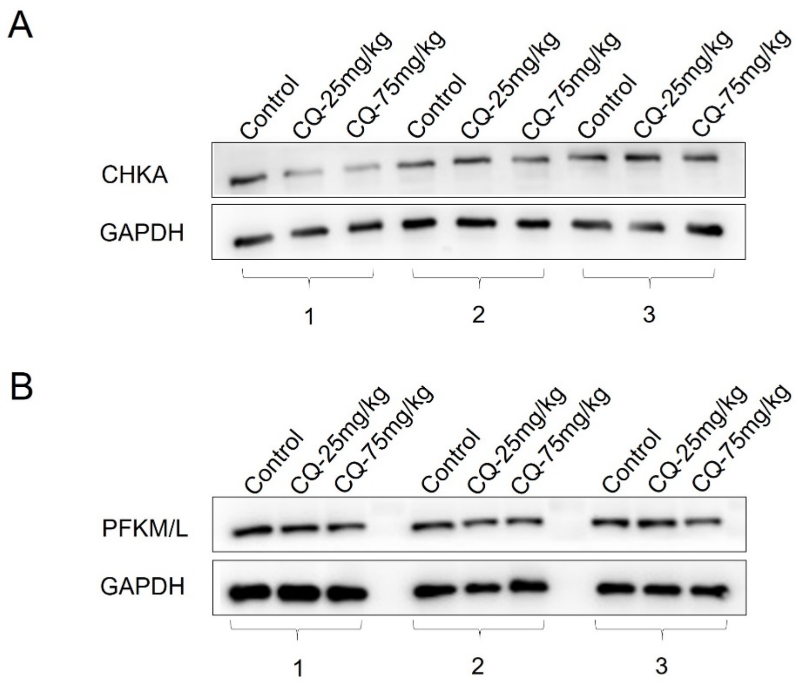

Figure S3. Western blot analysis of the expression level changes of CHKA (**A**) and PFKM (**B**) in tumor tissues after treatment with different doses of CQ, n = 3.

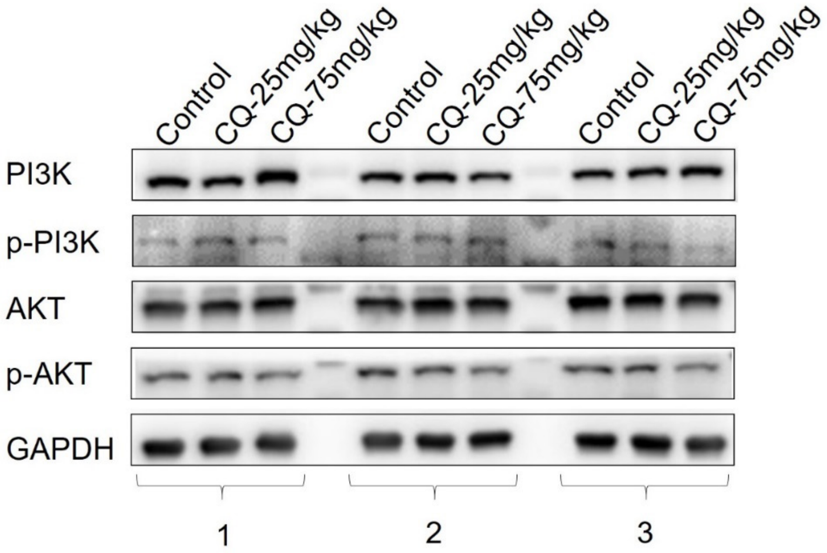

Figure S4. Western blot analysis of the expression level changes of key proteins in the PI3K/AKT signaling pathway after treatment with different doses of CQ *in vivo*, n = 3.

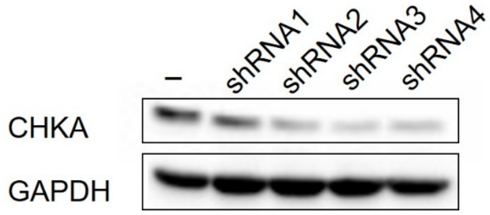

Figure S5. Western blot-based verification of the knockdown of CHKA proteins in Caco2 cells. Caco2 cells were transfected with lentiviral vectors containing different shRNA of CHKA, and the knockdown efficacy was examined by Western blot.
